# Characterization of Transcriptional, Epigenetic, and Phenotypic Plasticity and Discovery of Biomarkers in Acute and Chronic Murine Schistosomiasis Infection

**DOI:** 10.1096/fj.202502913R

**Published:** 2026-02-05

**Authors:** Sheila de Andrade Penteado Corrêa, Cauã da Silva Oliveira Teodoro, João Paulo Luz da Silva, Fabio Queiroz, Tayná Dandara do Amaral, Silmara Marques Allegretti, Fernanda Silva de Oliveira, Rafaela Ferraz Teixeira, Lizandra Maia de Sousa, Sílvio Roberto Consonni, Matheus de Souza Gomes, Christoph Grunau, Fernanda Janku Cabral

**Affiliations:** ^1^ Department of Animal Biology, Institute of Biology State University of Campinas (UNICAMP) Campinas Brazil; ^2^ Instituto Mario Penna Belo Horizonte Brazil; ^3^ Department of Biochemistry and Tissue Biology Institute of Biology, State University of Campinas (UNICAMP) Campinas Brazil; ^4^ Federal University of Uberlândia Patos de Minas Brazil; ^5^ IHPE, Univ Perpignan Via Domitia, CNRS, IFREMER, Univ Montpellier Perpignan France

**Keywords:** biomarkers, epigenetics, host–parasite relationship, murine host, next‐generation sequencing, *Schistosoma mansoni*

## Abstract

Parasites can induce changes in their hosts, favoring the success of the infection and its development at each stage of their life cycle. The host minimizes the effects of the parasite's presence through its defense system, balancing the parasite–host relationship. The intricate parasite–host relationship provides physiological, immunological, and molecular cues that suggest interaction and mutual regulation of the transcriptome and epigenome, promoting phenotypic plasticity and survival in a changing environment. There has been a growing interest in the epigenetic mechanisms of *Schistosoma mansoni* , a parasite with remarkable phenotypic plasticity in response to signals from the environment and its hosts. Several studies emphasize the epigenetic mechanisms behind the phenotypic plasticity of *Schistosoma*. Regarding the host's gene expression in the face of infection, however, there is little evidence of which pathways are altered by the passage of the parasite through the lungs and by the pathogenesis in the hepatic portal system. In this work, we characterized *S. mansoni* infection in parasitological and biochemical aspects of the murine model in comparison with the profile of the initial, acute, and chronic phases of infection (3, 7, and 20 wpi (weeks postinfection), respectively). The biochemical and morphological results of the infection at 3, 7, and 20 wpi show the phenotypic changes of schistosomiasis in the murine model. ATAC‐seq (Assay for Transposase‐Accessible Chromatin using sequencing) at 7 wpi shows a chromatin with higher accessibility for infected individuals, and Western blotting at 7 wpi shows an increase in histone marks H3K9ac and H3K9me3, indicating a change in chromatin status after infection. RNA‐seq (RNA sequencing) for 7 wpi results show a differential profile of lipid metabolism genes that are negatively modulated, while immune system genes are positively modulated. It is interesting to note that the negative modulation of mRNA expression of lipid pathway genes causes the rates of these metabolites to appear decreased in the blood, while the increased expression of immune system defense genes is in accordance with liver histology data, which shows an inflammatory profile.

## Graphical abstract

Workflow for discovering parasitological and molecular changes related to *Schistosoma mansoni* infection.

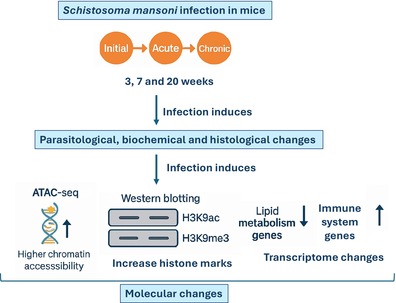

## 1 Introduction

The most intimate environment for parasites is the host. Once they have penetrated their host, they manipulate its morphology, behavior, and microbiota, creating an environment favorable to their survival; in response, the host triggers its complex defense system [1, 2, 3]. Parasites also modulate host gene expression by epigenetic mechanisms from a biochemical‐molecular arsenal capable of activating and/or repressing gene transcription, regulating cellular processes, and leading to the recruitment of protein complexes during transcription [4, 5].

There has been a growing interest in the epigenetic mechanisms of *Schistosoma mansoni* , the etiological agent of schistosomiasis. This parasite presents great phenotypic plasticity in response to signals from the aquatic environment—to which the larval stages miracidia and cercaria are subjected—and from its hosts—mollusks of the genus *Biomphalaria* and humans and/or rodents. Such signs favor the success of the infection and the development of the parasite in each phase of its life cycle [6].

In the vertebrate host, young parasites circulate through the lungs, but the liver and intestines are the microenvironments in which adults lodge and produce hundreds of eggs a day, half of which are retained in these organs. This induces a vigorous granulomatous inflammatory response, followed by fibrosis that occurs with the participation of cell adhesion molecules and the matrix substrate, and promotes a change in the expression and environment of cytokines [7, 8]. In this way, there is a balance in the parasite–host relationship, because while it protects the host's intestine from infections by other pathogens and destroys miracidia, it facilitates the transit of parasite eggs in the intestine and their exit through the feces. This intricate parasite–host relationship provides physiological, immunological, and molecular cues that suggest interaction and mutual regulation of the transcriptome and epigenome that enable them to produce phenotypic plasticity and survive in a changing environment [9, 10].

Considering that recent data related to the epigenetics of *S. mansoni* indicate that the progression of its development is related to histone modifications and to the activity of histone‐modifying enzymes, we hypothesize that, in the presence of the parasite, there is a remodeling of the host's epigenome and transcriptome by induction of biochemical pathways in the host's liver, provoking alteration in the expression of transcription factors and regulation of factors related to the stress response.

Preliminary unpublished studies involving Chromatin Immunoprecipitation (ChIP) showed that histone PTMs can be modulated by infection in order to alter the expression of cytokines in the host's liver. This suggests that a large part of the vertebrate host's epigenome can be altered, evidencing its plasticity in schistosomiasis (personal communication).

Our research group also demonstrated that the parasite alters its reproductive fitness when chromatin proteins of the parasite have their expression decreased through RNA interference. This was evidenced through the infection of the mammalian host by parasites that had the expression of the HP1 gene decreased by RNAi [11], indicating that epigenetic modifications are important for the progression of the life cycle of the parasite and schistosomiasis disease.

However, our knowledge regarding epigenetic changes in host immunity to infection is still limited, which warrants further investigation. Experiments performed using mice with knockout for immunological receptor have shown that the alteration of immunological background influences epigenetic in liver tissues and parasitological features in the parasite [12]. In addition, considering that epigenetic mechanisms are viable targets for therapy due to their dynamic and reversible nature, as has been observed in cancerology [13, 14, 15, 16], we believe that understanding the changes that occur in the epigenome of the murine host in response to the parasite would provide a significant improvement in schistosomiasis treatment strategies.

Thus, we aim to perform parasitological and molecular characterization of the epigenome and transcriptome of the murine model of experimental infection of *S. mansoni* by investigating the effects of acute and chronic infection of the mammalian host to identify the genes and epigenetic features affected by pathogenesis in the liver. We hope that these results will enable advances in the treatment of schistosomiasis and the vaccination of the population in the coming decades.

## 2 Materials and Methods

### 2.1 Experimental Murine Host Infection

Cercariae of *S. mansoni* were obtained from *B. glabrata* snails kept at the Department of Animal Biology of UNICAMP. 30‐day‐old female Balb/c mice, weighing about 25 g, were mechanically contained and individually infected by the tail immersion technique in cercarial suspension (≈70 cercariae), for 2 h with exposure to light, as described by Oliver & Stirewalt [17]. Infected and non‐infected animals were kept in the house of Parasitology (DBA‐UNICAMP) under the same conditions, with water and ad *libitum feed*. All procedures were carried out in accordance with the recommendations of the Ethics Committee for the Use of Animals (CEUA/UNICAMP, protocol No. 6150‐1/2022).

### 2.2 Experimental Design

The animals were organized into three experimental groups of 5 animals each (*n* = 5): (1) initial infection (3 weeks); (2) acute infection (7 weeks); (3) chronic infection (20 weeks). For each infection period evaluated, there will be a control (CTRL) group consisting of uninfected animals.

### 2.3 Parasitological Characterization of the Acute Phase of the Experimental Model

Infected mice and the CTRL group were weighed weekly. From the 3rd week post‐infection (wpi), the feces of the infected animals were collected for the Kato‐Katz test to quantify the number of eggs eliminated in the feces. After the infection period of each experimental group (3, 7, and 20 wpi), all mice were euthanized by cervical displacement. The infected animals were submitted to perfusion of the hepatic portal system and mesenteric veins, according to the protocol of Pellegrino & Siqueira [18], for worm recovery. A fragment of the small intestine of the infected animals was collected for an oogram. This examination makes it possible to determine the number of eggs of each stage of maturity (1st to 5th and dead eggs) that are retained in this tissue, according to the protocol as previously described [19]. Biochemical tests were performed on blood samples to evaluate the liver profile of infected and non‐infected mice. The serum of each animal was collected and processed in the A25 Biosystems automated system of the veterinary clinical analysis laboratory VETPAT (Campinas‐SP). The liver and spleen of each animal, infected or not, were collected and weighed. Fragments of the lower region of the right lobe of the liver were fixed in buffered 10% formaldehyde and destined for histological analysis. Another 50 mg fragment of the liver was frozen for chromatin extraction, next‐generation sequencing assay, and placed in RNA later (Invitrogen) for RNA extraction (RNA‐seq).

### 2.4 Histopathological Examination

Specimens of the hepatic tissue of all mice were fixed in buffered 10% formaldehyde for 24 h for fixation, followed by washing under flowing water. After washing, they were dipped in ethyl alcohol in increasing concentrations, cleared and purified in xylene, and inserted in paraffin wax. Each block of paraffin was segmented into sections of 4–6 μm, and then 5 parts were removed for Hematoxylin & Eosin [20], and Masson's trichrome staining [21].

### 2.5 ATAC‐Seq and RNA‐Seq of the Liver

ATAC‐Seq was done as described in (https://wellcomeopenresearch.org/articles/5‐121). Briefly, 50 mg of liver fragment was washed once with 50 μL of cold PBS buffer, and all supernatant was removed with a pipette. Next, we add 50 μL of prepared transposase mixture with 25 μL of 2× TD buffer (Illumina, 0.5 μL 1% IGEPAL (Sigma‐Aldrich), and 22 μL of molecular biology grade water). The samples were homogenized with a small piston and pipetted 10 times to break up the tissue. To fragment the Tn5 accessible chromatin and label the DNA with adaptor sequences, 2.5 μL of TDE1 (Illumina) was added, and the samples were incubated at 37°C with 300 rpm agitation for 30 min. Subsequently, the chromatin was purified using the QIAquick PCR Purification kit, and the purified DNA was eluted in 20 μL of elution buffer (Tris–HCl 10 mM, pH 8). Next, the DNA of each sample was nick‐repaired and amplified by PCR using NEBNext High Fidelity mix according to the method described previously [22]. The PCR cycles were as follows: cycle of 72°C for 5 min, 98°C for 30 s, 5 cycles of 98°C for 10 s, 63°C for 30 s, 72°C for 1 min. The additional number of PCR reaction cycles was determined by qPCR on 10% of the amplification reaction to which SYBR had been added. The fragments were then separated by electrophoresis in a 1.5% agarose gel for visual inspection. Purification through AMPure XP beads was performed to remove small fragments according to the protocol described previously [22]. NGS was performed on a NextSeq 550 at the BioEnvironnement sequencing facility of the University of Perpignan Via Domitia as paired‐end 150 bp reads. Reads were processed on the French Galaxy instance (https://usegalaxy.fr). Quality was checked with FastQC, Adapter trimming performed with Trim Galore!, and alignment with Bowtie2 on the mouse genome mm10, evoking a sensitive end‐to‐end option. PCR duplicates were removed with rmdup. Peak‐calling was executed with MACS2 callpeak using both infected and uninfected liver, identifying 6799 regions. ChromstaR, a Hidden‐Markov‐Model‐based tool, identified 8682 regions of ATAC enrichment. We used the bedtools Intersect intervals to count reads in the individual Bowtie2 BAM files overlapping MACS peak regions, and DESeq2 to identify significant differences at adjusted *p* < 0.05. ChromstaR analysis was done with default parameters. Metagene profiles over peak regions were generated by removing background signal with MACS bgdcmp using the MACS‐generated BedGraph treatment and BedGraph control files and using this as input into DeepTools [23, 24, 25, 26, 27].

For the RNA‐seq experiments, the total RNA extracted with the SV RNA kit (Promega)s was analyzed for its concentration using the Qubit RNA High Sensitivity kit (Invitrogen) and read in the Qubit 3.0 (ThermoFischer); and for quality, using the RNA sensitivity kit (Agilent) in the Tapestation 4200 equipment (Agilent), according to the manufacturer's instructions. The sequencing libraries were built using the TruSeq Stranded mRNA (Illumina) kit as instructed by the manufacturer. The concentration of the libraries was measured using Qubit (Invitrogen), and the quality was analyzed using TapeStation 4200 (Agilent). The libraries were denatured and sequenced in the NovaSeq X (Illumina) equipment using the 10B 2 × 150 cycles kit, according to the manufacturer's instructions. The quality analysis of the generated sequences was performed using the FastQC tool [28]. Low‐quality readings (Phred score ≤ 30) and adapters were filtered using the Trimmomatic tool [29]. The remaining sequences were reanalyzed with FastQC to confirm the required quality score. Finally, they were mapped and aligned to the reference genome (GRCm39) using the STAR software [30]. The alignment parameters “outFilterMultimapNmax 1” and “quantMode GeneCounts” were included to generate uniquely mapped reads and count the number of reads mapped to each specific gene. The reads were filtered with HTSFilter [31] and normalized with DESeq2 [24]. Differential gene expression (DGE) analyses were performed using DESeq2 and visualized using heatmaps using the Pheatmap (version 1.0.12) of R [32]. Only genes with log2 fold change (Log2FC) > 1 or < −1 and adjusted *p*‐value (padj) < 0.05 were considered differentially expressed. Using bioinformatics resources, we created genetic libraries for infected and non‐infected animals.

### 2.6 Histone Extraction and Western Blotting

The histone extraction protocol was performed using the Abcam/ab113476 kit. Each liver sample was cut and transferred to the Dounce homogenizer, to which 500 μL of 1X pre‐lysis buffer was added. The homogenate was transferred to Eppendorf tubes, and these were centrifuged at 10000 rpm for 1 min at 4°C. After discarding the supernatant, the pellet was resuspended in 200 μL of 1X pre‐lysis buffer. Samples were kept on ice for 10 min to complete lysis of the cells and then centrifuged at 10000 rpm for 1 min at 4°C. The supernatant was discarded, the pellet resuspended in 200 μL of lysis buffer, and the tubes were incubated on ice for 30 min. A new centrifugation at 12000 rpm for 5 min at 4°C was performed, and the supernatant, containing the proteins, was transferred to new tubes and, to each of them, 150 μL of balance‐DTT buffer was added. The protein concentration was measured with a Bradford assay. A 20 μL sample of each sample was diluted in 10 μL of water and applied to the electrophoresis gel and submitted to Western blotting.

## 3 Results

### 3.1 Phenotypical Characterization of Acute and Chronic Phases of the Murine Model: Parasitological, Biochemical, and Histological

To characterize the biological model of infection as well as the phenotype of hosts to compare with the transcriptome and epigenome, we measured parasitological parameters of infection such as animal body weight, liver and spleen weight, number of worms and eggs, and oogram, which estimates the number of viable eggs (mature, immature, and dead eggs). For the three periods studied, we found that both infected animals and those in the control group generally showed a statistically significant increase in body weight over the weeks (Figure S1).

**FIGURE 1:**
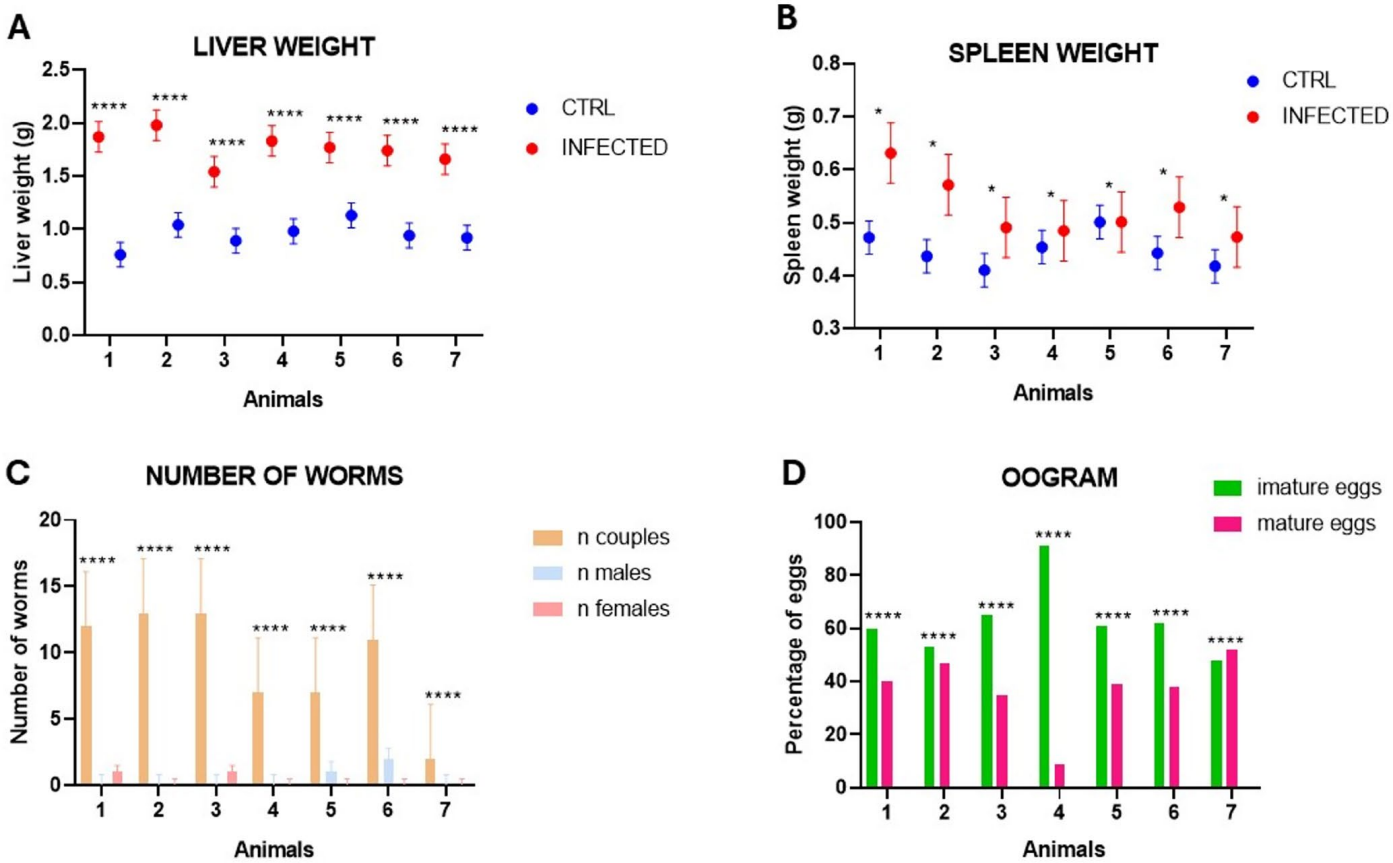
Parasitological analyses for the phenotypical period of 7 wpi. (A) Comparison of the infected animals' liver weight and the control group (CTRL). (B) Comparison of the infected animals' spleen weight and the control group (CTRL). (C) Number of worms (pairs, males, and females) recovered after perfusion. (D) Comparison of the maturity stages of the eggs retained in the intestine of each animal. (****) Indicates *p* < 0.0001; (***) *p* = 0.0009 and (*) *p* < 0.01—Two‐way ANOVA.

**FIGURE 2:**
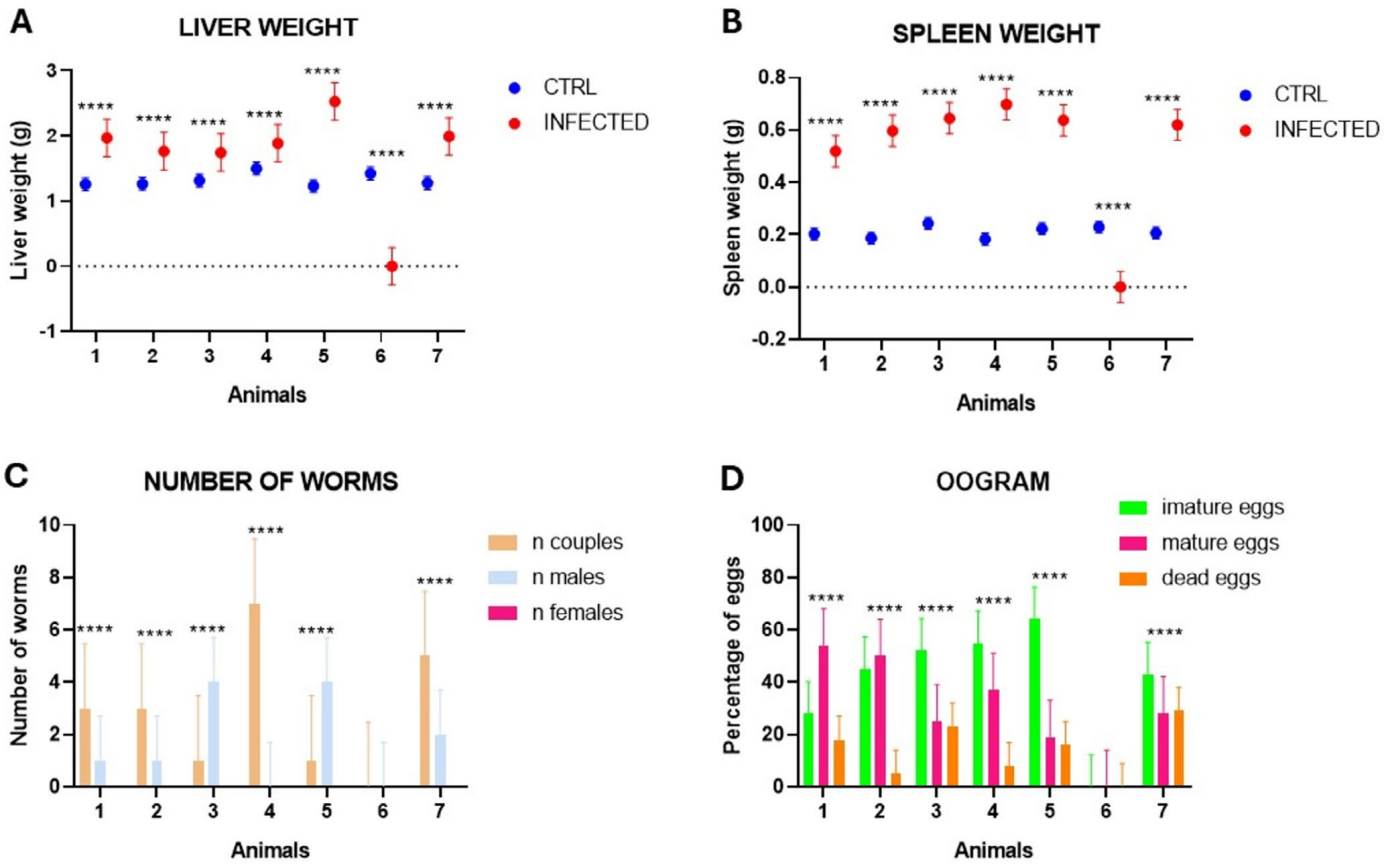
Parasitological analyses for the phenotypical period of 20 wpi. (A) Comparison of the infected animals' liver weight and the control group (CTRL). (B) Comparison of the spleen weight of infected animals, infected animals' spleen weight (g), and the control group (CTRL). (C) Number of worms (pairs, males, and females) recovered after perfusion. (D) Comparison of the maturity stages of the eggs retained in the intestine of each animal. (****) Indicates *p* < 0.0001; (**) *p* = 0.0095 and (*) *p* < 0.01—Two‐way ANOVA.

Regarding liver weight, no statistically significant difference was observed between infected animals and the CTRL group only for the period of 3 wpi, and as expected, we do not have oviposition and worms after perfusion because oviposition occurs after male and mate pairing and copulation, which occurs after 7 weeks of infection (Figure S2A). However, spleen weight 3 wpi is statistically increased (Figure S2B). For 7 wpi, liver weight is statistically increased, supposedly because eggs presence (Figure 1A), while spleen weight has a tendency of increasing but it is not statistically significantly increased in the acute phase of infection (Figure 1B). Our data has shown that at 7 wpi clearly marks the increase of number of couples (Figure 1C) and eggs also increased (Table S1), which is reflected in the oogram (Figure 1D). As expected, the number of immature eggs is statistically higher, reflecting clearly the status of acute infection and host response on eggs maturation in acute schistosomiasis (Figure 1D). For 20 wpi, infected animals showed a statistically significant increase in the size and weight of the liver and spleen, evidencing the dependence of immune response of the host (Figure 2A,B). However, the number of worms in couples (Figure 2C) reduced statistically compared with 7 wpi. But surprisingly, the number of mature eggs depends on the individual, reflecting the dependence of the individual features of host response and infection for egg maturation in chronic schistosomiasis (Figure 2D).

**FIGURE 3:**
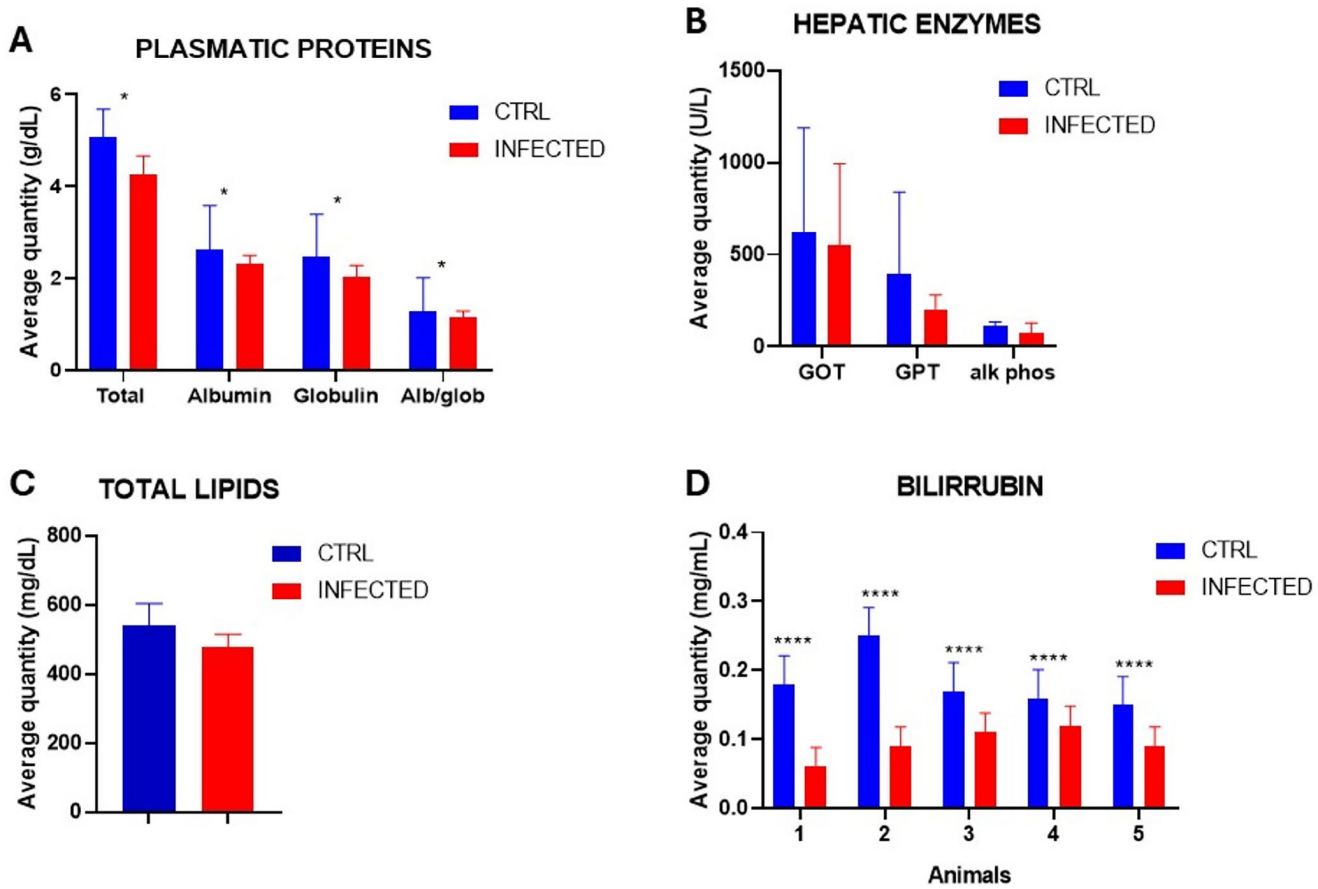
Results of the biochemical analyses for the period of 7 wpi. (A) Plasma protein values of each animal in the control group (CTRL) and the infected group. (B) Comparison of the mean value of each plasma protein from infected animals and CTRL. (C) Liver total lipid values of infected animals and CTRL. (D) Comparison of the mean amount of bilirubin of infected animals and CTRL. (***) Indicates *p* = 0.0095 and (*) *p* < 0.01—Two‐way ANOVA.

**FIGURE 4:**
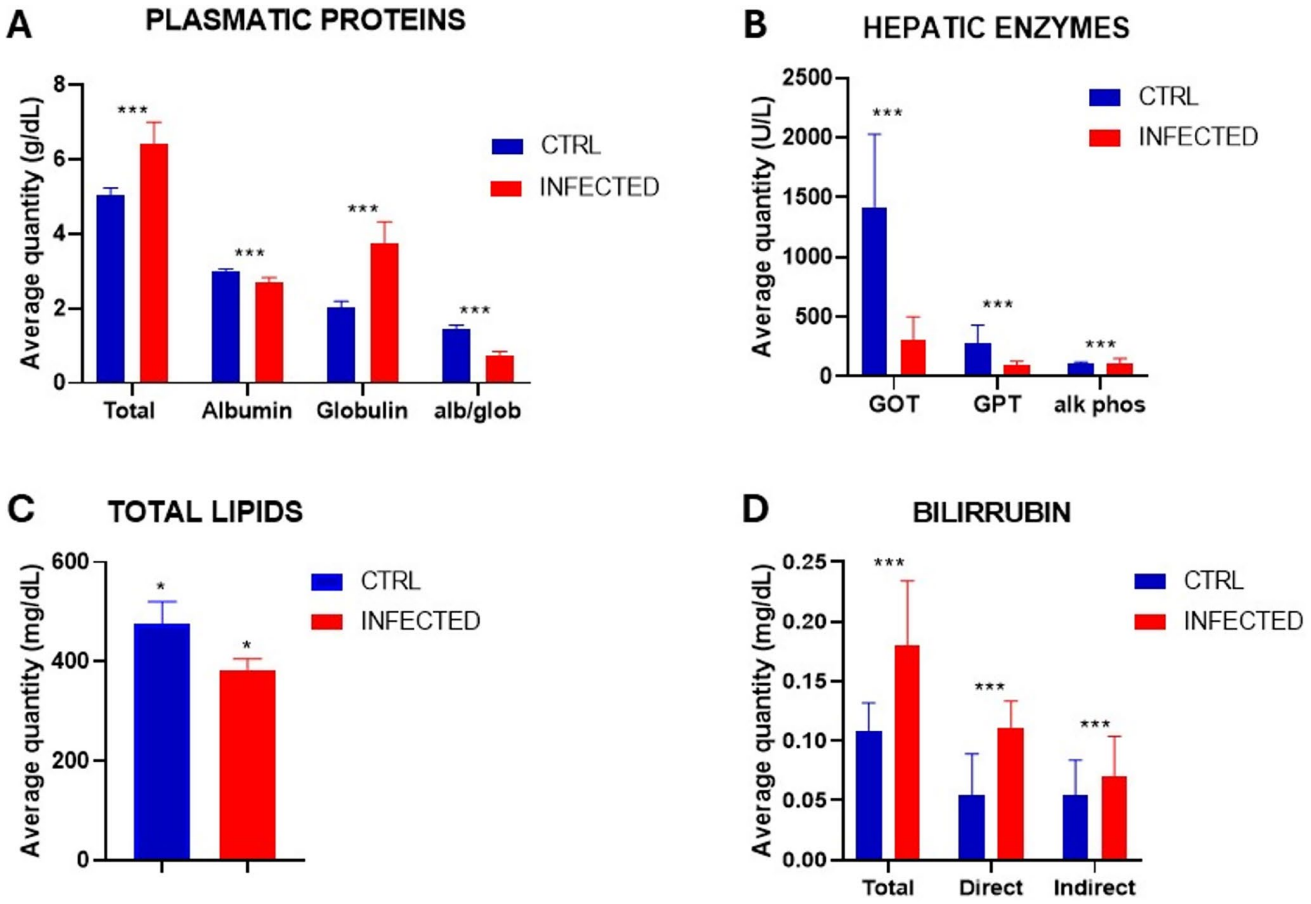
Results of blood biochemical analyses for the period of 20 wpi. (A) Plasma protein values for each animal in the control group (CTRL) and the infected group. (B) Comparison of the mean value of each liver enzyme of infected animals and CTRL. (C) Liver lipid values of infected animals and CTRL. (D) Comparison of the mean amount of bilirubin of infected animals and CTRL. (***) Indicates *p* = 0.0095 and (*) *p* < 0.01—Two‐way ANOVA.

Another important parameter evaluated in the phenotypical characterization of acute and chronic schistosomiasis was the biochemical parameters of plasma protein content, activity of hepatic enzymes, amount of bilirubin, and total lipid content. The blood of 7‐wpi and 20‐wpi mice was collected, evaluated and presented in Figures 3 and 4. Unfortunately, it was not possible to collect blood for the animals at 3 wpi because the amount collected was insufficient for the analysis. The difficulty of collection was attributed to the small size of the mice during this period.

The difference in plasma protein values between infected animals and the control group was statistically significant at 7 and 20 wpi. At 7 wpi, the animals showed a reduction in the levels of total proteins, albumin, and globulin (Figure 3A). The levels of total lipids and enzymes—Aspartate Aminotransferase (GOT), Glutamic‐Pyruvic Transaminase (GPT), and Alkaline Phosphatase (Alk Phosh) of infected animals and the control group were not statistically significant for 7wpi animals (Figure 3B,C). In contrast, the data for bilirubin levels have diminished at 7 wpi infected compared with CTRL and were statistically significant (Figure 3D).

The chronic disease at 20 wpi revealed a change in the profile of content of protein in the plasma with a statistically significant reduction in globulin levels and an increase in total proteins and albumin (Figure 4A). However, animals infected at 20 wpi showed a significant decrease in concentration in the plasma of liver enzymes and total lipids (Figure 4B,C) and an increase in bilirubin (Figure 4D).

For the histological profile, liver sections were also submitted to histological analyses. Figures S3–, S5 show temporal differences in infection between non‐infected (CTRL) and infected tissues. Our results agree with the histological profile of acute and chronic disease already described [9].

### 3.2 RNA‐Seq and ATAC‐Seq of the Acute Disease of the Experimental Schistosomiasis Model

In the face of phenotypical parameters observed in our study, we decided to choose the acute phase to perform RNA‐seq and ATAC‐seq. The acute 7wpi was chosen due to its high inflammatory profile, marked presence of parasite and eggs, and some statistically significant alteration of blood biochemistry. For this, total RNA was extracted from liver fragments obtained from infected animals and CTRLs. The results obtained through the Tapestation (Agilent) presented in the figure (Figure S6) showed that the RNAs were intact.

**FIGURE 5:**
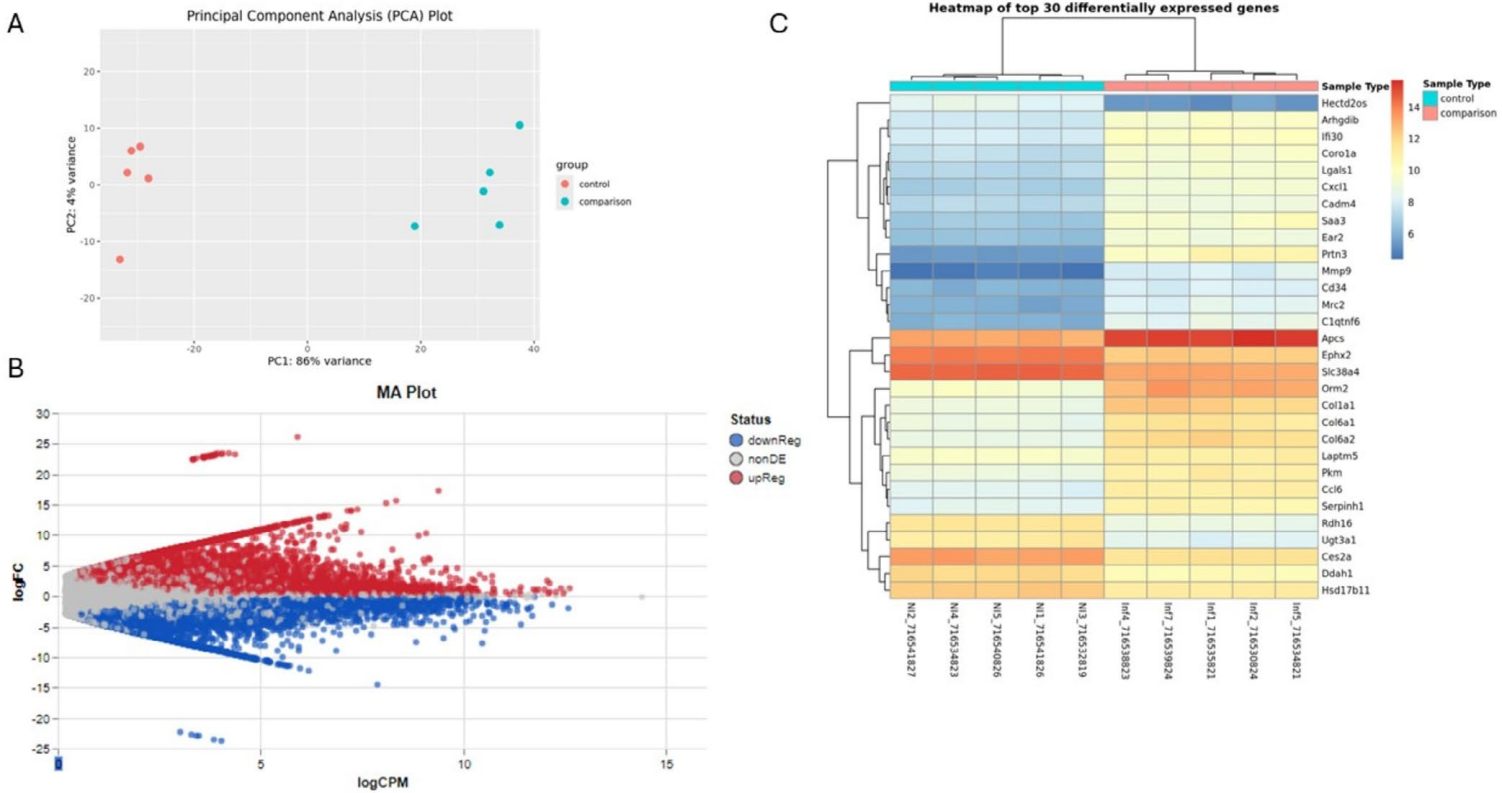
Heatmap showing the RNA‐seq result of the uninfected animals (CTRL) and those infected with 7 wpi. (A) Principal component analysis showing the differences between CTRL and infection (comparison). (B) Volcano plot for the sequenced genes with differential expression from the RNA of each animal. (C) Heatmap for the 30 sequenced genes with differential expression.

The RNA‐seq analysis for the 7 wpi is shown below (Figure 5) and in the Supporting Information. The principal component analysis data (Figure 5A) show the grouping of the gene samples according to their variation. Our results show that the genes expressed in infected animals are distinct from those expressed in control animals. This is confirmed in the volcano plot, which shows the genes with increased and decreased expression (Figure 5B), and in the cluster analysis for the 30 genes with alteration in expression, as shown in Figure 5C.

**FIGURE 6:**
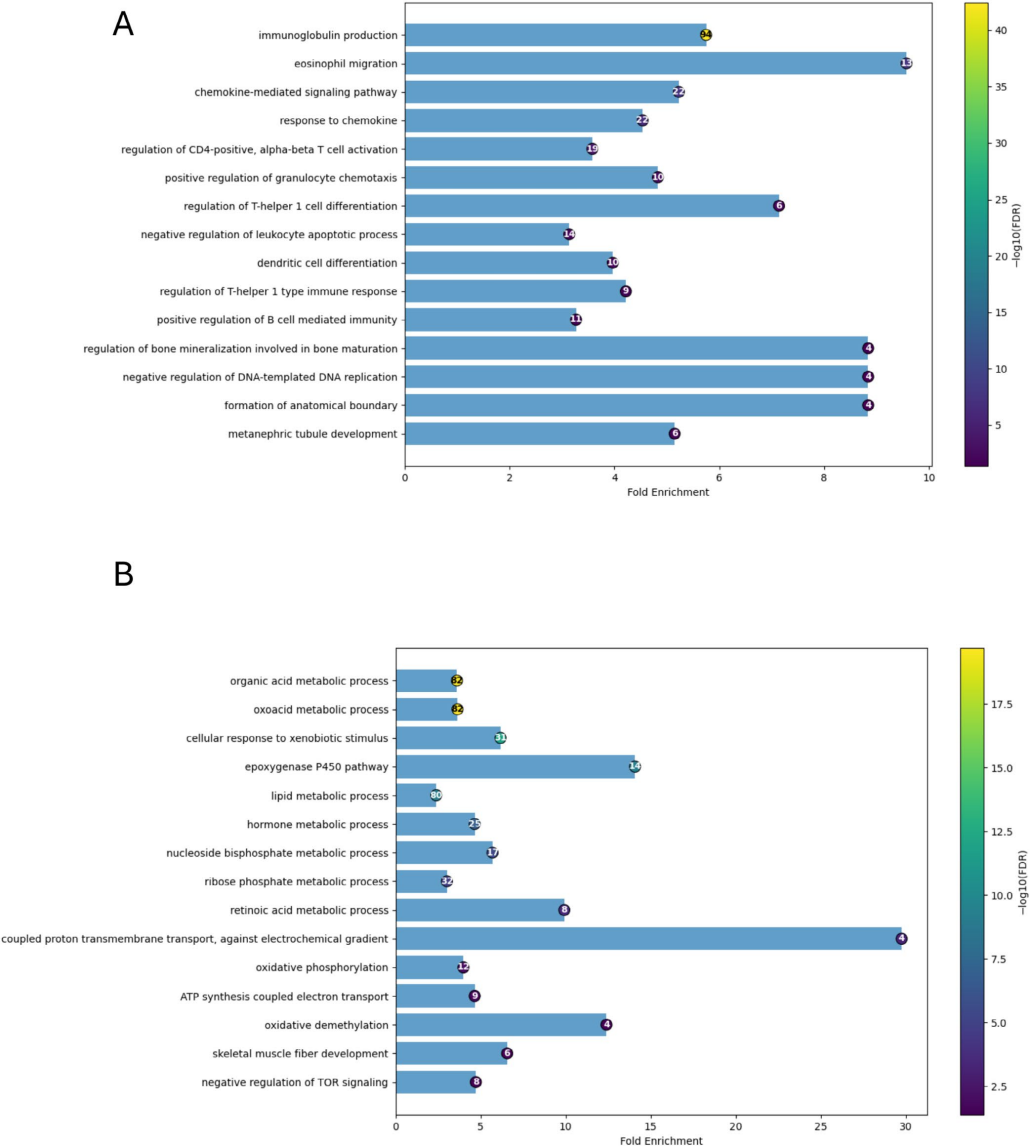
Bar chart of significantly overrepresented GO Biological Process terms (FDR < 0.05). The horizontal bars represent the fold enrichment ranked by increasing false discovery rate (FDR). GO terms were aggregated using Gene Ontology semantic similarity based on the GO graph structure (Resnik‐based semantic similarity), where a representative (based on the lowest FDR) was selected for each semantic cluster. The circles at the end of each bar have their size proportional to the number of GO terms grouped within each semantic cluster, while circle color represents statistical significance expressed as −log10(FDR). The color scale of the −log10(FDR) is shown in a vertical bar on the right. The numbers highlighted inside the circles indicate the number of genes associated with the representative GO term. The plot is organized to show the most significant terms in the top portion of the chart. (A) Up‐regulated genes; (B) Down‐regulated genes.

With the RNA‐seq data, we also performed Gene Ontology analyses. The results are shown in Figure A, for the most expressed genes, and in Figure 6B, for the least expressed genes. The results in Figure 6A clearly show that genes for immune defense are highly expressed, while genes for lipid metabolism decrease (Figure 6B). This indicates that the parasite modulates the expression of the host transcriptome by decreasing the expression of lipid pathway genes, which results in a decrease in the amount of total lipids in the blood at 7 wpi (Figure 3), and this decrease in total lipids becomes more critical at 20 wpi of infection (Figure 4). The high expression of genes encoding components of the immune system is fully consistent with the morphological changes in the liver tissue shown in Figures S4 and S5, where established granulomas and evident areas of inflammatory infiltrate are evident.

By bringing together the analysis of parasitological, histological, transcriptomic analysis, and GO for the transcripts sequenced, we found 8 genes that are candidates for biomarkers of murine schistosomiasis. These genes are: Prtn3, Orm2, Col1a1, Col6a1, Col6a2, Pkm, Ccl6, Serpinh1. The function of these genes is described in the Table S2. Briefly, these genes are related to metabolic processes, immunity, and acute hepatic diseases. Prtn3 is related to tissue degradation, which is related to the tissue processes in schistosomiasis, as shown in Figures S3–, S5. In addition, Col genes and Serpinh1 are related to fibrosis, which we can correlate with our histology data. Furthermore, Orm2, Pkm, and Ccl6 are related to acute hepatic disease. Orm2 is particularly interesting because it is secreted by hepatocytes, regulates hepatic lipogenesis, and we found a modulation of lipids described in Figures 3 and 4. In addition, the ORM protein is reduced in NAFLD patients, reducing cytokine production, modulating the immunological system, which is typical of schistosomiasis, where the parasite may modulate the immunological system to gain the advantage of the survivor itself and the host [9].

**FIGURE 7:**
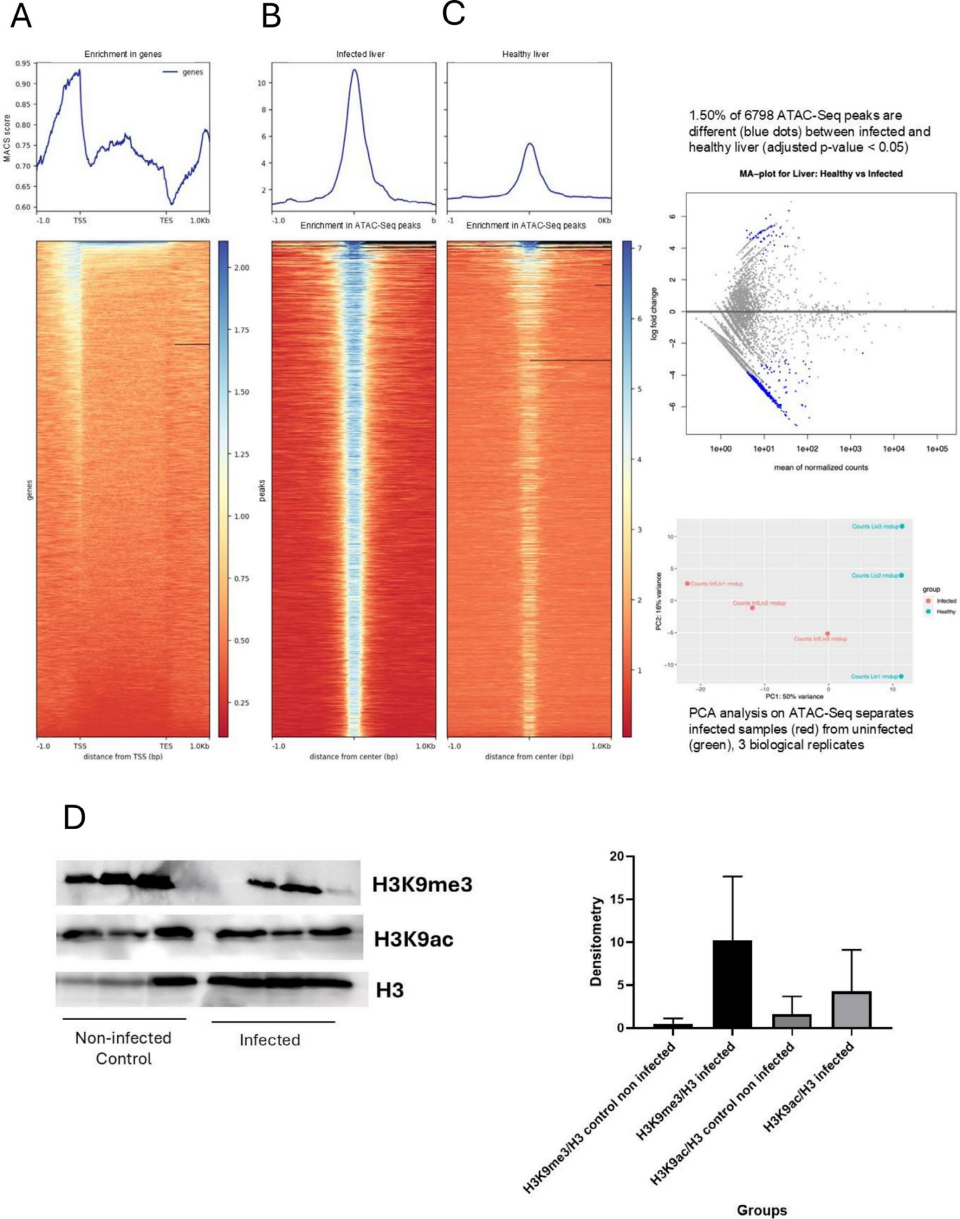
Result of the analysis of ATAC‐seq of (A) liver chromatin, (B) infected, and (C) healthy (CTRL) animals. (D) Western blotting for the post‐translational modifications H3K9me3 and H3K9ac in infected and uninfected animals (CTRL). Histones were extracted and subjected to Western blotting with anti‐H3K9me3 and anti‐H3K9ac antibodies.

To elucidate the chromatin profile in infected animals at 7 weeks of infection, an assay of ATAC‐seq was performed to compare the chromatin profiles of the livers of three infected and three healthy animals (CTRL). A statistically significant chromatin pattern was observed between the groups analyzed (Figure 7). DESeq2 identifies 102 differentially Tn5 accessible regions (1.5% of the 6799 total ATAC‐Seq peaks), and ChromstaR detects 1584 regions (18.2% of 8682 total). Interestingly, average ATAC‐Seq profiles over all peaks are indicative of a higher accessibility of chromatin in infected livers (Figure 7A–C). This is in line with the differential peak detection of ChromstaR, which finds 1464 regions that are Tn5 accessible in infected but not in healthy liver, but only 121 that occur in healthy liver vs infected liver.

In addition to chromatin analysis by ATAC‐seq, we extracted the histones and analyzed them by Western blotting, using anti‐H3K9me3 and anti‐H3K9ac antibodies. The results of Western blotting are shown in Figure 7D and indicate that methylation (H3K9me3) and acetylation (H3K9ac) marks are increased in infected animals when compared to healthy animals (CTRL). These results strongly support the fact that infection leads to chromatin structure modulation, which can be associated with a transcriptome modulation (Figure 6), resulting in the phenotype changes described in the infection periods shown in this study (Figures 1, 2, 3, 4, 5, 6, 7).

## 4 Discussion

The acute phase of schistosomiasis (also called fever or Katayama syndrome) occurs between the 2nd and 8th week post‐infection and corresponds to the period of the parasite's migration in the host organism until the beginning of egg laying by the adult worms. It can be characterized by fever, skin lesions (rash, urticaria), pulmonary symptoms (cough, dyspnea), and eosinophilia—symptoms most frequently observed in tourists and immigrants who are in endemic areas [33].

The chronic phase is characterized by a progressive increase in oviposition after the 8th wpi. About half of these eggs are released into the feces, and half are lodged in the small venules of the liver, lung, and intestine, stimulating the influx of immune cells that leads to the development of a granulomatous reaction. While they protect the host and destroy the eggs, they cause fibro‐obstructive disease, hepatomegaly, and portal hypertension [34]. Subsequently, hepatic blood flow is altered, resulting in increased flow and stasis in the collateral venous system, especially in the splenic portal territory. This event triggers greater retention of blood cells with consequent development of splenomegaly [35].

In the period of 7 wpi, we observed a marked increase in the number of eggs released in the feces. This period corresponds to the phase in which the worms that have recently arrived from the lung mature pair, migrate to the mesenteric plexus capillaries, and begin laying eggs [36]. Before this period, we found a slight presence of immature eggs or their absence in the feces of the animals. Another study describes the release of immature eggs in the period of 4–5 wpi by newly mated worms. We found, through the oogram, many eggs retained in the tissues, and the most significant portion corresponded to immature eggs. No dead eggs were verified. The predominance of immature eggs in the oogram indicates that oviposition is still active and continuous [37].

The comparison of the spleen weight of the infected animals with the control group revealed a small degree of significant difference (*p* < 0.01). This data may indicate an incipient increase in the activity of this organ producing inflammatory cytokines, mainly IL‐4, IL‐5, and IL‐13, which control the process of granuloma formation. According to a previous study [38], up to the period of 8 weeks post‐infection, the immune response is mild compared to chronic infection. Unpublished results from our research group showed high expression of IL‐4 cDNA, but lower expression for IL‐5 and IL‐13.

In addition, we found a statistically significant difference in the body weight of infected animals in relation to those in the control group. The effect of schistosome infection typically reduces or maintains the body weight of mice unchanged, except when metabolic disorders are present. Studies conducted with obese mice have shown an increase in the number of worms and mature eggs, demonstrating that the parasite modulates the host's lipid metabolism to increase its survival and reproduction [39].

The liver is an important metabolic organ, containing enzymes that play important roles in the metabolism of endogenous and exogenous substances. For this reason, we collected blood samples from infected mice and CTRL and plotted the liver's metabolic profile.

TGO (oxaloacetic transaminase) and TGP (pyruvic transaminase), also called, respectively, AST (aspartate aminotransferase) and ALT (alanine aminotransferase), are intracellular enzymes present in large quantities in hepatocytes. TGP is mainly found in the cytoplasm of the hepatocyte, while 80% of AST is present in the mitochondria. Injury to or destruction of liver cells releases these enzymes into the circulation. In mild hepatocellular damage, the predominant form in serum is TGP, while in severe lesions, AST is released, increasing the TGO/TGP ratio [40]. Thus, the dosage of these enzymes in the blood reflects the functioning status of the liver. Elevated TGO and TGP concentrations indicate inflammation of the liver. Abnormal elevation in alkaline phosphatase levels is observed in nearly all liver disorders and is associated with bile flow obstruction [41]. Increased values of these enzymes have been found in patients infected with *S. mansoni* . During the 7 weeks, we observed that the infected animals exhibited changes in the concentration of liver enzymes TGO, TGP, and alkaline phosphatase, which were not considered significant when compared to the control group.

The decrease in the concentration of total proteins and plasma albumin fits the profile of acute inflammatory responses. Globulin concentration in this phase can be variable (decreased, normal, or increased). We observed a statistically significant reduction in the concentration of plasma proteins (total, albumin, globulin) in the serum of infected animals in relation to CTRL. Similar results were observed by other researchers [42] and [41].

Bilirubin is the substance produced by the liver after metabolizing the hemoglobin released in the destruction of senescent erythrocytes. It performs several essential functions in the body. In addition to being an indicator of liver health, it also acts as an antioxidant, protecting cells from damage caused by free radicals. In addition, bilirubin is involved in regulating the metabolism of fats and carbohydrates and the absorption of fat‐soluble vitamins such as vitamins A, D, E, and K [43]. Abnormal amounts of this substance in plasma are indicative of liver problems such as biliary lesions or obstructions. Abebe et al. [42] found a positive correlation between increased plasma bilirubin concentration and more eggs in the feces. Xue et al. [44] demonstrated the relationship between increased plasma bilirubin concentration and liver fibrosis in the chronic phase in mice infected with *S. japonicum* . We showed a significant increase in the bilirubin concentration of the infected animals in relation to the CTRL group at 7 wpi, the period that precedes the chronic phase of schistosomiasis.

According to another study [45], abnormalities in lipid metabolism are commonly seen in the hepatosplenic form of schistosomiasis, notably decreased levels of total cholesterol, IRT, and HDL. Experimental studies have shown that reduced total cholesterol levels in infected mice can be explained by the fact that *S. mansoni* cannot synthesize the cholesterol necessary for egg growth and production [46, 47]. Da Silva et al. [48] also demonstrated decreased plasma lipid levels in patients infected with *S. mansoni* . However, our analysis did not confirm significant changes in total lipid concentration over the 7‐wpi period.

Studies have associated schistosome infection with the lower prevalence of metabolic diseases and diabetes in humans and rodents, whose effect can be partly attributed to an anti‐inflammatory profile of macrophages, but also to transcript reprogramming of genes related to phospholipid metabolism and glucose in liver macrophages [49]. Oviedo and colleagues demonstrated that schistosome infection may induce, through IL‐4, an increase in mitochondrial fatty oxidation by promoting innate immunological training focused on increasing mitochondrial fatty acid oxidation and consequent decrease in cholesterol in male hosts, but not in females. These data corroborate the results of RNA sequencing. The ORM2 gene is responsible for secreting proteins of equal name by hepatocytes, which act as a key regulator of liver lipogenesis and liver metabolic diseases, such as NAFLD (non‐alcoholic fatty liver disease), and immunity. We observed that this gene was overexpressed in the infected female mice but was expressed little in the female mice of the control group (not infected). Interestingly, plasma cholesterol levels were not reduced, which, according to previously described [50], is related to ovarian hormones produced by schistosome‐infected females that have a blocking effect on the metabolic change. Our biomarkers found here are promising molecules to improve schistosomiasis diagnosis through mapping the protein‐coding genes in the blood and plasma of the infected hosts. Further studies may be designed to first map these proteins on the murine host and then translate this research to human hosts.

Currently, epigenetics is a tool that has been used to unravel possible chromatin changes triggered by environmental factors. These alterations promote covalent modifications in histones, such as acetylation, deacetylation, methylation, demethylation, and ubiquitination [12].

In eukaryotic cells, the organization of the chromatin landscape modulates the accessibility of genomic regions and dynamic responses to external and internal stimuli, which represents an essential feature for gene regulation. In general, accessible genomic regions are enriched in regulatory elements important for gene activity, while inaccessible regions restrict the binding of transcriptional regulators, resulting in gene silencing [51]. Complex parasite–host relationships can lead to alterations in the regulation of the host's transcriptome, as well as modifications in the phenotype of its cells [52].

Our ATAC‐seq protocol is a simplified version of the transposase‐accessible chromatin assay with high‐throughput sequencing that positively displays chromatin‐accessible regions to Tn5 (open chromatin regions). Western blots on a limited number of histone modifications and our draft ATAC‐seq assay jointly portray the liver chromatin landscape of infected animals that clearly differ from that observed in animals in the CTRL group. We hypothesize that these changes lead to transcriptome modulation, resulting in the phenotype changes described in the infection period shown in this work. Further work is needed to establish causal relations between the epigenome, transcriptome, and phenotype of the experimental infection model correlated to parasitological observations.

Our data show, for the first time, the comparison of parasitological, morphological, and molecular landscapes. These data show us the complexity of schistosomiasis as an infectious disease and open space for more complex studies of this parasite–host relationship and its implications for the control, diagnosis, and treatment of *S. mansoni* .

## Author Contributions

S.A.P.C. designed the study, performed experiments, analyzed data, and wrote the manuscript; C.S.O.T. performed experiments; J.P.L.S. analyzed RNA‐seq data; F.Q. performed experiments; T.D.A. performed experiments; S.M.A. provided resources; F.S.O. performed experiments; R.F.T. performed experiments; L.M.S. performed experiments; S.R.C. provided resources; M.S.G. provided resources; C.G. provided resources, performed and analyzed ATAC‐seq experiments; F.J.C. designed the study, performed experiments, analyzed data, and wrote the manuscript.

## Funding

This work was supported by Fundação de Amparo à Pesquisa do Estado de São Paulo (FAPESP) (2021/14982‐6); Fundação de Amparo à Pesquisa da Universidade Estadual de Campinas (FAEPEX) (2169/23 and 2083/24).

## Conflicts of Interest

The authors declare no conflicts of interest.

## Supporting information


**Data S1:** fsb271457‐sup‐0001‐DatasetS1.xlsx.

**Figure S1:** fsb271457‐sup‐0002‐FigureS1.pptx.

**Figure S2:** fsb271457‐sup‐0003‐FigureS2.jpg.

**Figure S3:** fsb271457‐sup‐0004‐FigureS3.jpg.

**Figure S4:** fsb271457‐sup‐0005‐FigureS4.jpg.

**Figure S5:** fsb271457‐sup‐0006‐FigureS5.jpg.

**Figure S6:** fsb271457‐sup‐0007‐FigureS6.pptx.

**Table S1:** fsb271457‐sup‐0008‐TableS1.xlsx.

**Table S2:** fsb271457‐sup‐0009‐TableS2.xlsx.

## Data Availability

The data that support the findings of this study are available in BioProject ID PRJNA1314140.
