# Supplementary material for: Characterization of Transcriptional, Epigenetic, and Phenotypic Plasticity and Discovery of Biomarkers in Acute and Chronic Murine Schistosomiasis Infection
Source: FASEB J. 2026 Feb 5;40(3):e71457. doi: 10.1096/fj.202502913R (PMC12875175; doi:10.1096/fj.202502913R)
Supplement: Supplementary file 2 — Figure S1: fsb271457‐sup‐0002‐FigureS1.pptx. [file FSB2-40-e71457-s009.pptx]

## Slide 1
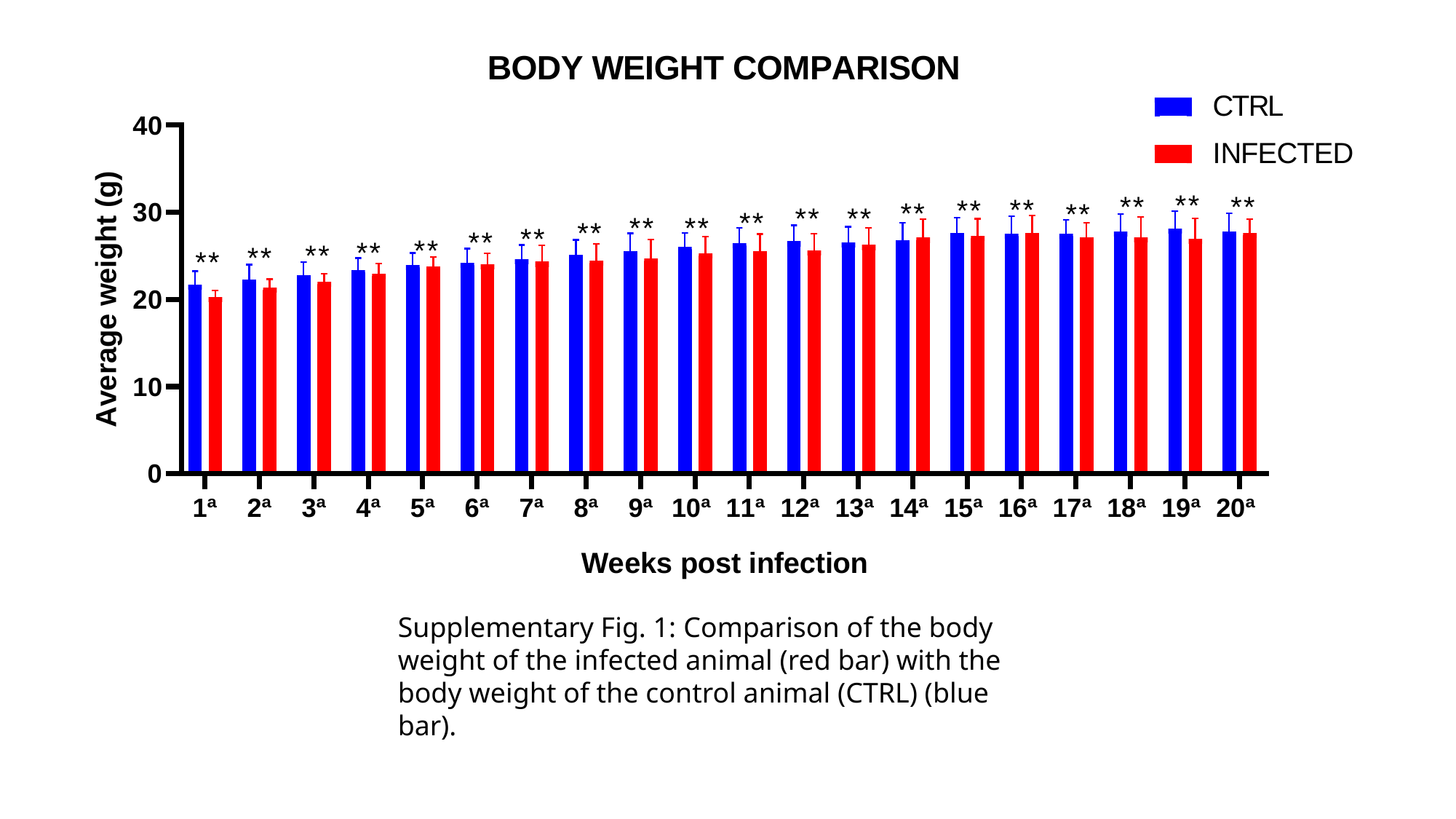

Supplementary Fig. 1: Comparison of the body weight of the infected animal (red bar) with the body weight of the control animal (CTRL) (blue bar).
