# Supplementary figures and images for: Characterization of Transcriptional, Epigenetic, and Phenotypic Plasticity and Discovery of Biomarkers in Acute and Chronic Murine Schistosomiasis Infection
Source: FASEB J. 2026 Feb 5;40(3):e71457. doi: 10.1096/fj.202502913R (PMC12875175; doi:10.1096/fj.202502913R)

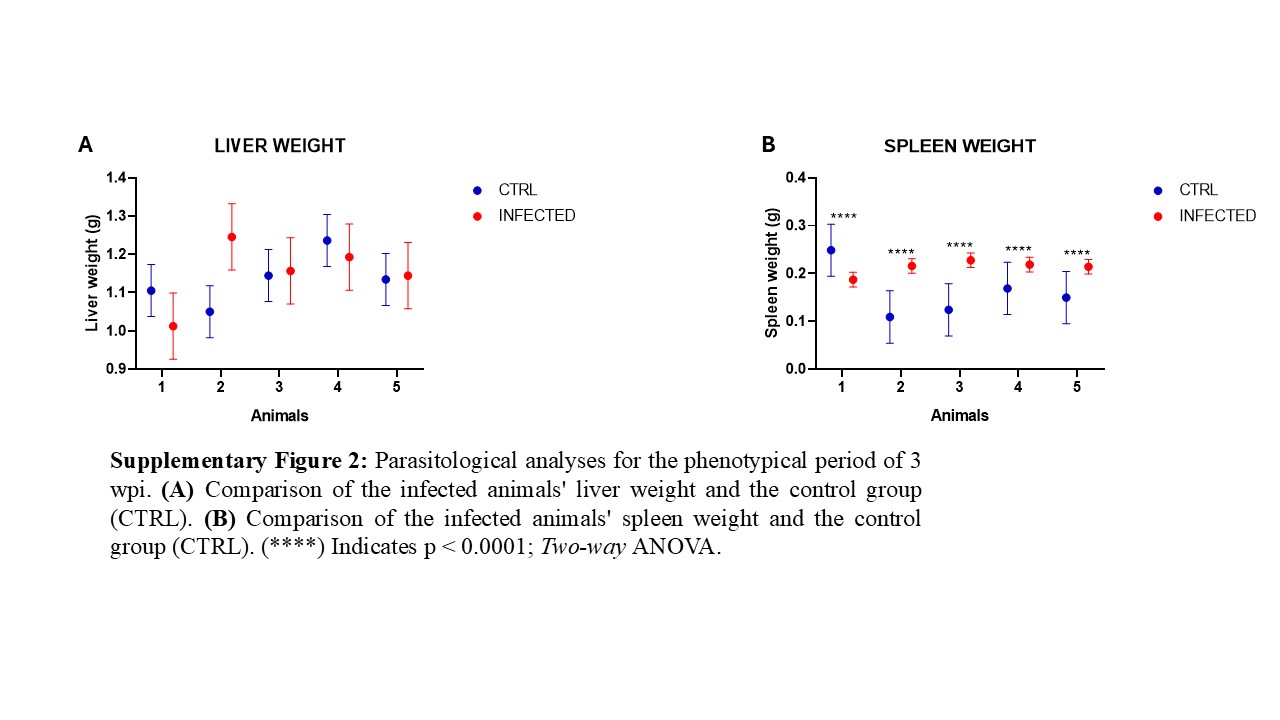

Supplement: Supplementary file 3 — Figure S2: fsb271457‐sup‐0003‐FigureS2.jpg. [file FSB2-40-e71457-s006.jpg]

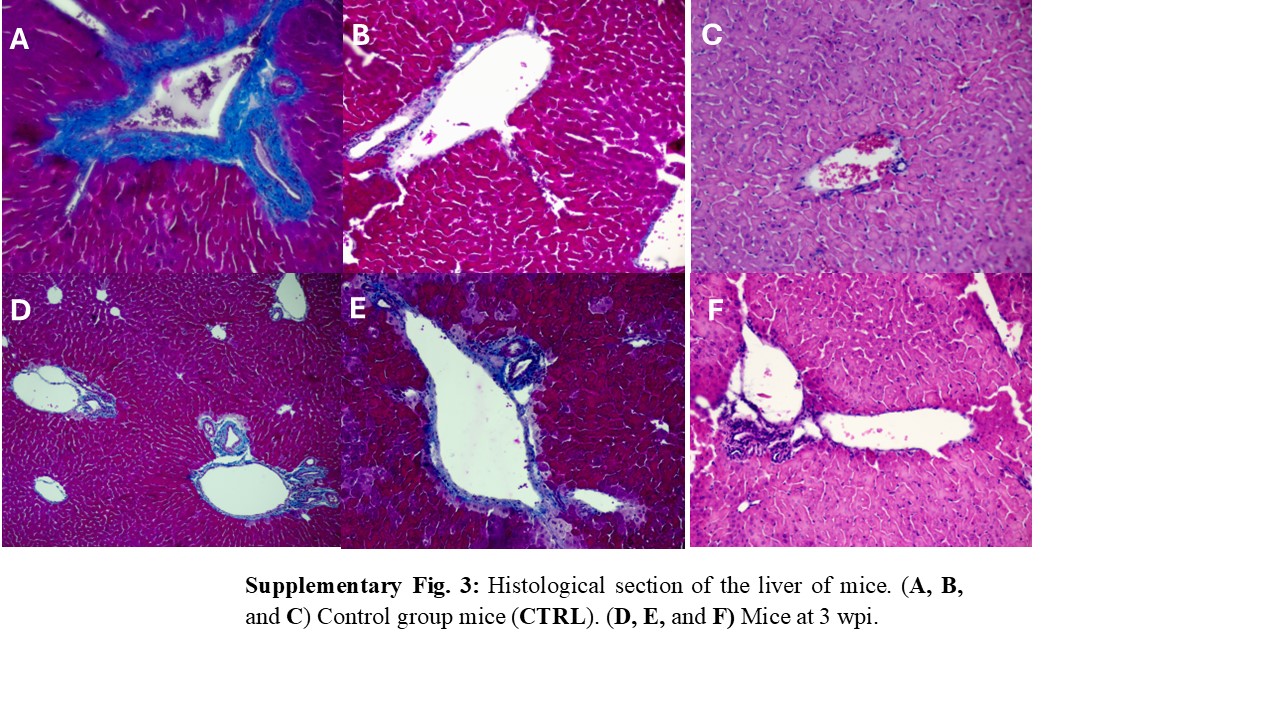

Supplement: Supplementary file 4 — Figure S3: fsb271457‐sup‐0004‐FigureS3.jpg. [file FSB2-40-e71457-s008.jpg]

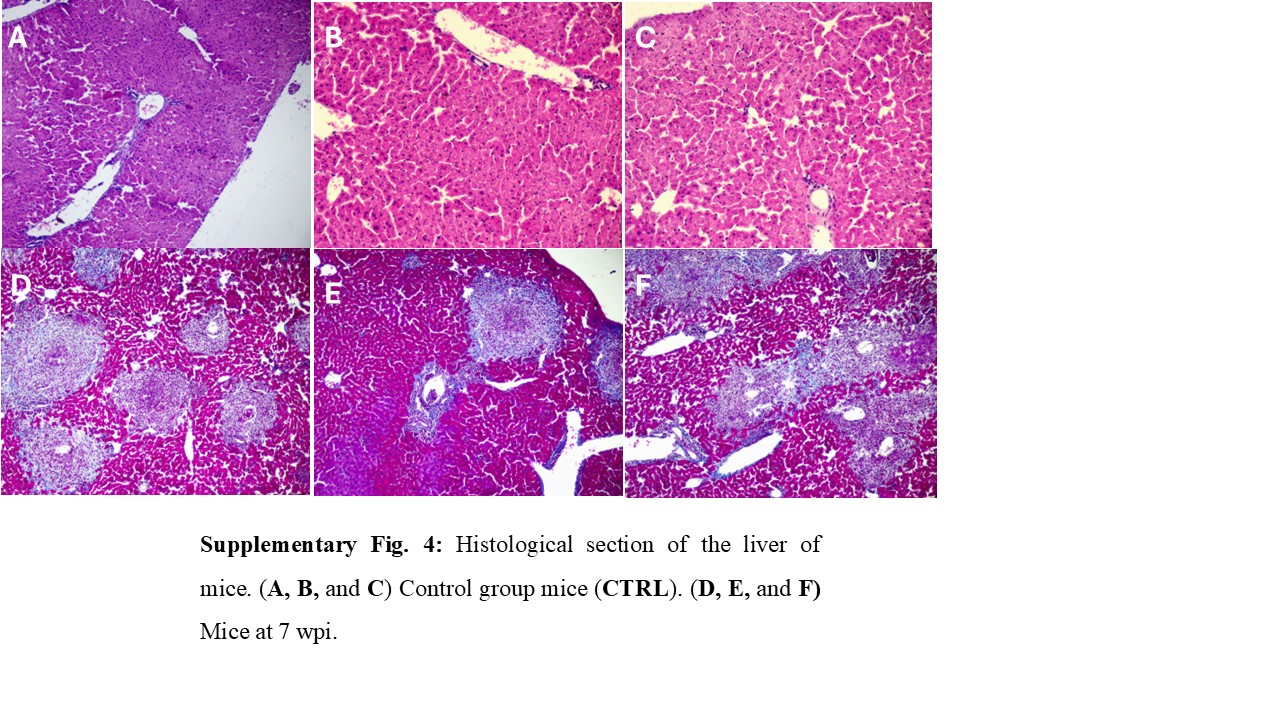

Supplement: Supplementary file 5 — Figure S4: fsb271457‐sup‐0005‐FigureS4.jpg. [file FSB2-40-e71457-s001.jpg]

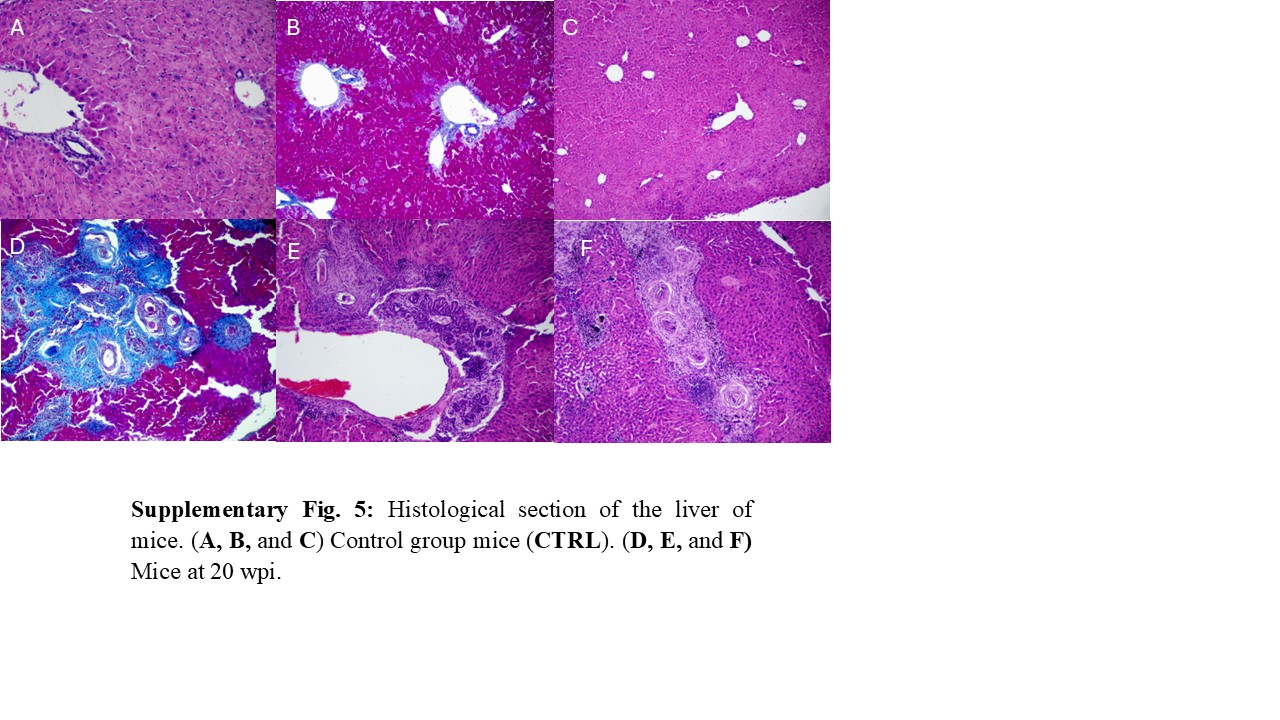

Supplement: Supplementary file 6 — Figure S5: fsb271457‐sup‐0006‐FigureS5.jpg. [file FSB2-40-e71457-s005.jpg]
