# Supplementary material for: Characterization of Transcriptional, Epigenetic, and Phenotypic Plasticity and Discovery of Biomarkers in Acute and Chronic Murine Schistosomiasis Infection
Source: FASEB J. 2026 Feb 5;40(3):e71457. doi: 10.1096/fj.202502913R (PMC12875175; doi:10.1096/fj.202502913R)
Supplement: Supplementary file 7 — Figure S6: fsb271457‐sup‐0007‐FigureS6.pptx. [file FSB2-40-e71457-s004.pptx]

## Slide 1
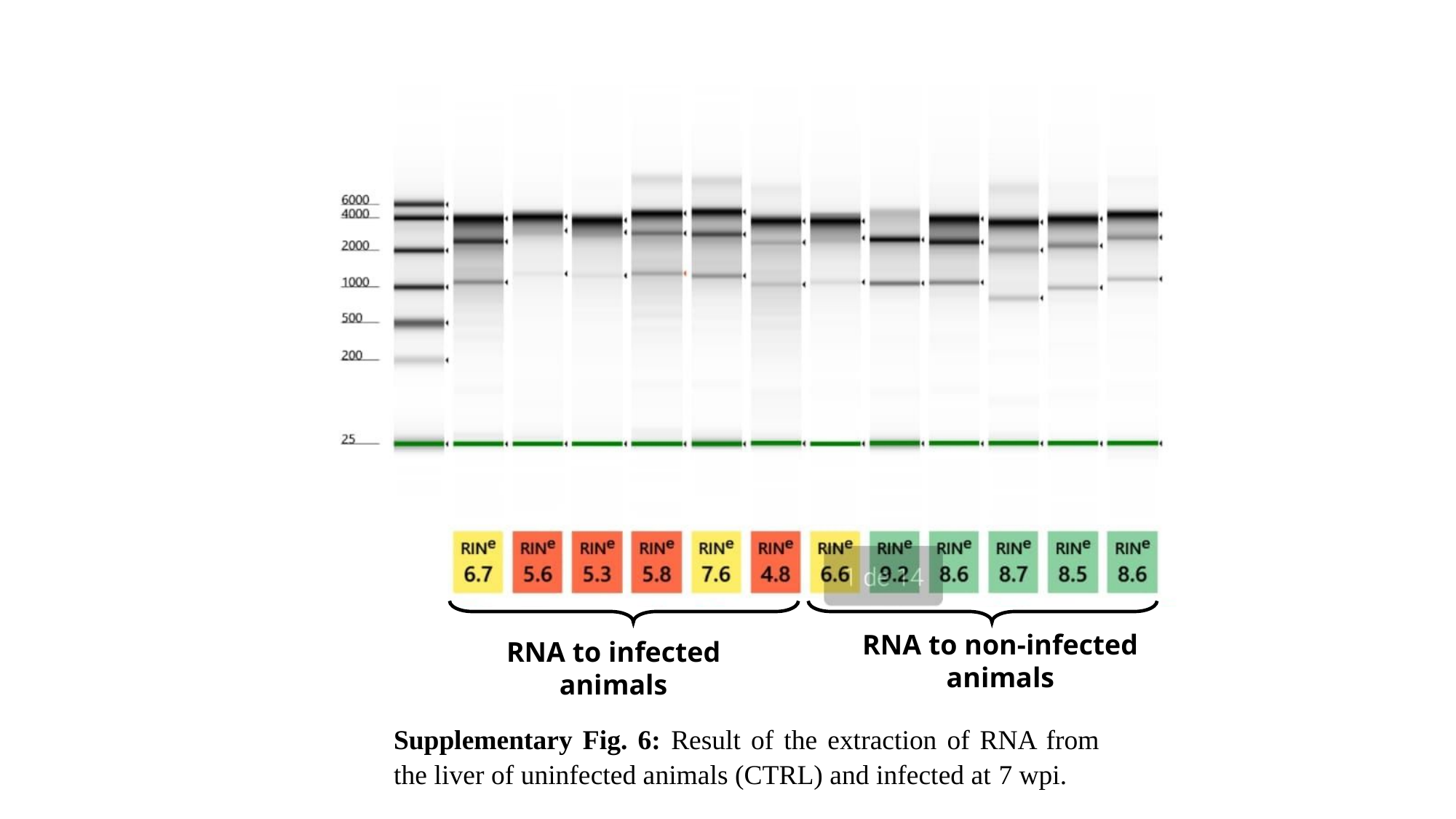

RNA to non-infected animals
RNA to infected animals
Supplementary Fig. 6: Result of the extraction of RNA from the liver of uninfected animals (CTRL) and infected at 7 wpi.
